# Potential and pitfalls of ^89^Zr-immuno-PET to assess target status: ^89^Zr-trastuzumab as an example

**DOI:** 10.1186/s13550-021-00813-7

**Published:** 2021-08-21

**Authors:** Marc C. Huisman, C. Willemien Menke-van der Houven van Oordt, Josée M. Zijlstra, Otto S. Hoekstra, Ronald Boellaard, Guus A. M. S. van Dongen, Dhaval K. Shah, Yvonne W. S. Jauw

**Affiliations:** 1 Department of Radiology and Nuclear Medicine, Amsterdam UMC, Vrije Universiteit Amsterdam, Cancer Center Amsterdam, De Boelelaan 1117, 1081 HV Amsterdam, The Netherlands; 2 Department of Medical Oncology, Amsterdam UMC, Vrije Universiteit Amsterdam, Cancer Center Amsterdam, Amsterdam, The Netherlands; 3 Department of Hematology, Amsterdam UMC, Vrije Universiteit Amsterdam, Cancer Center Amsterdam, Amsterdam, The Netherlands; 4 Department of Pharmaceutical Sciences, School of Pharmacy and Pharmaceutical Sciences, The State University of New York at Buffalo, Buffalo, USA

**Keywords:** ^89^Zr-immuno-PET, Modelling, Molecular imaging, Monoclonal antibody, Target expression

## Abstract

**Background:**

^89^Zirconium-immuno-positron emission tomography (^89^Zr-immuno-PET) is used for assessment of target status to guide antibody-based therapy. We aim to determine the relation between antibody tumor uptake and target concentration to improve future study design and interpretation.

**Methods:**

The relation between tumor uptake and target concentration was predicted by mathematical modeling of ^89^Zr-labeled antibody disposition in the tumor. Literature values for trastuzumab kinetics were used to provide an example.

**Results:**

^89^Zr-trastuzumab uptake initially increases with increasing target concentration, until it levels off to a constant value. This is determined by the total administered mass dose of trastuzumab. For a commonly used imaging dose of 50 mg ^89^Zr-trastuzumab, uptake can discriminate between immunohistochemistry score (IHC) 0 versus 1–2–3.

**Conclusion:**

The example for ^89^Zr-trastuzumab illustrates the potential to assess target expression. The pitfall of false-positive findings depends on the cut-off to define clinical target positivity (i.e., IHC 3) and the administered mass dose.

## Introduction

^89^Zr-immuno-PET is used in clinical studies as a non-invasive method to quantify tumor uptake of ^89^Zr-labeled monoclonal antibodies (mAbs). A potential application is to non-invasively assess target expression in-vivo. If reliable, this knowledge may help to decide whether individual patients may benefit from drugs aimed at this target. However, unexplained false-positivity occurs [1], hampering clinical applications.

An example is the use of ^89^Zr-trastuzumab to assess human epidermal growth factor receptor 2 (HER2) expression in-vivo. The goal is to predict which patients are likely to respond to treatment with trastuzumab or trastuzumab-based antibody–drug conjugates. Ideally, tumor uptake on PET should be high for HER2-positive tumors (IHC 3, 2/fluorescence in situ hybridization (FISH) amplified), and low for HER2-negative tumors (IHC 0, 1, 2/FISH non-amplified).

Our aim is to use mathematical modelling of antibody disposition in the tumor to demonstrate how tumor uptake of ^89^Zr-immuno-PET is related to the target concentration.

This general method will be illustrated using ^89^Zr-trastuzumab as an example to improve our understanding of false-positive findings.

## Materials and methods

We developed a mathematical model to predict tumor uptake [in terms of standardized uptake value (SUV)] as a function of time. Here, we first define the biological processes involved in mAb extravasation and target interaction. Next, we describe the parameters used to model these processes. Finally, we describe implementation of the model.

### Biological processes

**Fig. 1:**
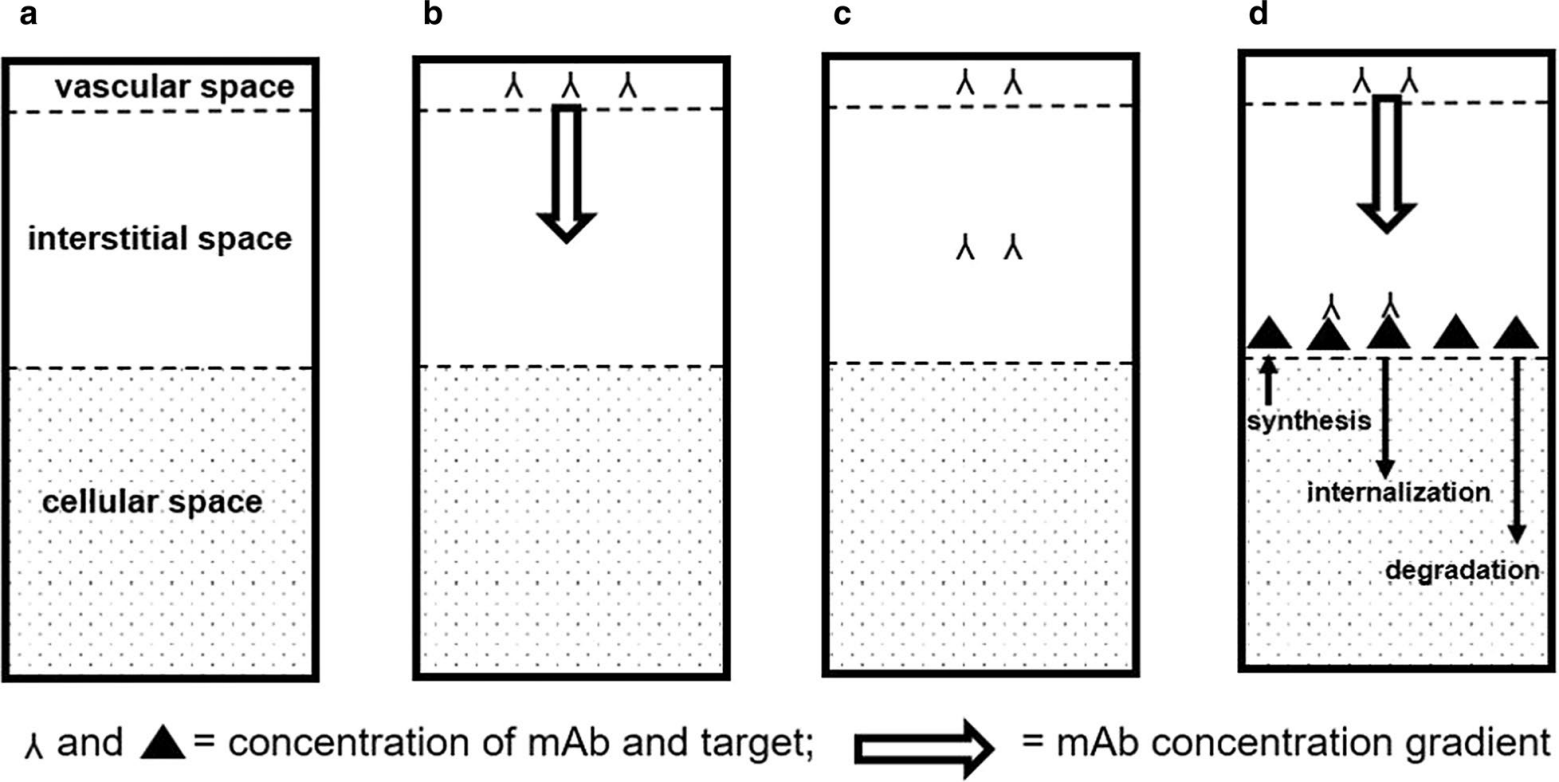
Tumor uptake of mAbs. Tumor volume fractions (**a**). Distribution of mAb after administration (**b**). Equilibrium distribution of mAb in a tumor without target expression (**c**). Distribution of mAb in a HER2-positive tumor (**d**). Target can be synthesized and degraded, mAb-target complexes can be internalized

Tumor tissue consists of vascular, interstitial and cellular spaces (Fig. 1a). Immediately after intravenous administration, all mAb is present in the vascular space (Fig. 1b). mAb extravasates as the concentration of unbound mAb in interstitial space is lower than the concentration of mAb in vascular space, with the concentration gradient as the driving force of extravasation. The extravasation rate constant determines the amount of mAb that can extravasate for a given concentration gradient. This rate constant combines the permeability of the vasculature and the degree of vascularization of the tissue. Thus, mAb is transported from vascular to interstitial space until the concentrations of mAb in the vascular and interstitial space are equal (Fig. 1c). When mAb binds to target, the concentration of unbound mAb in interstitial space decreases, resulting in an increased amount of extravasating mAb (Fig. 1d). The concentration of targets available for binding is determined by the initial target concentration as well as target kinetics.

### Parameters

The parameters used to describe tumor uptake are given in Table 1. They include tumor tissue characteristics (vascular and interstitial volume fractions and the extravasation rate constant) and target-related parameters (initial target concentration, equilibrium binding affinity and target synthesis, degradation and mAb-target internalization rates). To obtain typical values for these parameters, a literature search was performed. Per parameter, its value is given together with the literature references on which this value is based (see Table 1). The remaining parameters include those used to characterize systemic plasma PK. Our description of plasma pharmacokinetic (PK), needed in the model to drive tumor uptake, is in the form of a 2 compartment model.

**Table 1:** Model parameters

Parameter	Abbreviation	Unit	Value	References
Tumor tissue				
Plasma volume fraction	pvf	**–**	0.04	[9, 10]
mAb accessible interstitial volume fraction	ivf	**–**	0.2	[9, 11, 12]
Extravasation rate constant	*k*_ev_	h^−1^	0.012	[13, 15]
Target—HER2				
Initial target concentration	*T*_0_	nM	0–2700^1^	[4, 14]
Equilibrium binding affinity	*K*_*D*_	nM	5	[14]
Target synthesis rate	*k*_syn_	nM· h^−1^	*k*_deg·_*T*_0_	[14, 16]
Target degradation rate	*k*_deg_	h^−1^	0.19	[14, 16]
Target-mAb internalization rate	*k*_int_	h^−1^	0.19	[14, 16]
Input				
Injected Dose of ^89^Zr-mAb in plasma	ID_*l*_	mg	3	[4]
Injected Dose of mAb in plasma	ID_*u*_	mg	47	[4]
Exchange rate (plasma to normal tissue)	*k*_1_	h^−1^	0.0722	[4, 5]
Exchange rate (normal tissue to plasma)	*k*_2_	h^−1^	0.1366	[4, 5]
mAb clearance rate from plasma	*k*_cl_	h^−1^	0.0083	[4, 5]
Fixed				
Plasma volume (fixed at 3 L)	*V*_*p*_	L	3	
Tumor volume (fixed at 1 mL)	*V*_*t*_	L	0.001	
Body weight (fixed at 80 kg)	BW	kg	80	
Calculated				
Target concentration	*T*	nM		
Amount of (^89^Zr-)mAb in plasma	*X*_*p,l*_ and *X*_*p,u*_	nmole		
Amount of (^89^Zr-)mAb in remainder of body	*X*_Rob,*l*_ and *X*_RoB,*u*_	nmole		
Amount of (^89^Zr-)mAb in interstitial space	*X*_*i,l*_ and *X*_*i,u*_	nmole		
Fraction of (^89^Zr-)mAb unbound in ivf	*f*_*u*_	–		
Amount of residualized ^89^Zr	*X*_*r*_	nmole		
Concentration of ^89^Zr in the tumor	*C*_*t*_	nM		
Standardized uptake value	SUV	–		

^1^The relation between IHC score and HER2 concentration has been described in literature: IHC 0 < 30 nM, IHC 1 = 30–120 nM, IHC 2 = 120–590 nM, IHC 3 > 590 nM [14]. These numbers reflect estimated expression levels before therapy, when target synthesis and degradation are in balance [14, 16]

### Model implementation

We assume that:

- The biological processes and all parameters apply the same to unlabeled mAb as well as ^89^Zr-mAb
- The combined concentration of mAb and ^89^Zr-mAb in the interstitial space determines the fraction of antibody bound to target [2]
- The intracellular concentration of ^89^Zr does not decrease after internalization and metabolization of a ^89^Zr-mAb-target complex, since it is a residualizing radionuclide [3]

**Fig. 2:**
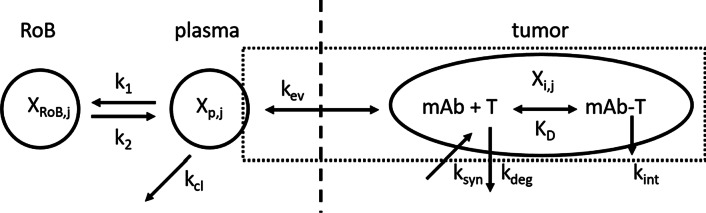
Schematic structure of the mathematical model. The area within the dotted line contains the contributions to the measured PET signal. The dashed line indicates the border between vascular and interstitial space. On the left hand side of the dashed line a two-compartment plasma PK model characterizes systemic PK. The subscript *j *can be either *l* (labeled) referring to ^89^Zr-mAb or *u* (unlabeled) referring to mAb

The structure of the mathematical model is shown in Fig. 2. In the model equations the subcript *j* refers to either unlabeled mAb (*j* = *u*) or labeled ^89^Zr-mAb (*j* = *l*).

The amount of (^89^Zr-)mAb is plasma is:

1 $$\frac{{{\text{d}}X_{p,j} }}{{{\text{dt}}}} = - \left( {k_{1} + k_{{{\text{cl}}}} } \right) \cdot X_{p,j} + k_{2} \cdot X_{{{\text{RoB}},j}} - k_{{{\text{ev}}}} \cdot \left( {\frac{{X_{p,j} }}{{V_{p} }} \cdot V_{t} - \frac{{f_{u} \cdot X_{i,j} }}{{{\text{ivf}}}}} \right), \,{\text{initial}} \,{\text{condition}}\, X_{p,j} = {\text{ID}}_{j}$$

The amount of (^89^Zr-)mAb in remainder of body is:

2 $$\frac{{{\text{d}}X_{{{\text{RoB}},j}} }}{{{\text{dt}}}} = k_{1} \cdot X_{p,j} - k_{2} \cdot X_{{{\text{RoB}},j}} ,\,{\text{ initial}} \,{\text{condition}}\, X_{{{\text{RoB}},j}} = 0$$

The amount of (^89^Zr-)mAb in the interstitial space of the tumor is:

3 $$\frac{{{\text{d}}X_{i,j} }}{{{\text{dt}}}} = k_{{{\text{ev}}}} \cdot \left( {\frac{{X_{p,j} }}{{V_{p} }} \cdot V_{t} - \frac{{f_{u} \cdot X_{i,j} }}{{{\text{ivf}}}}} \right) - k_{{\text{int}}} \cdot \left( {1 - f_{u} } \right) \cdot X_{i,j} , \,{\text{initial}} \,{\text{condition}}\, X_{i,j} = 0$$

The fraction unbound (^89^Zr-)mAb in the interstitial space of the tumor is calculated as:

4 $$f_{u} = 1 - \frac{{\left( {K_{D} + T + \frac{{\left( {X_{i,l} + X_{i,u} } \right)}}{{{\text{ivf}} \cdot V_{t} }}} \right) - \sqrt {\left( {K_{D} + T + \frac{{\left( {X_{i,l} + X_{i,u} } \right)}}{{{\text{ivf}} \cdot V_{t} }}} \right)^{2} - 4 \cdot \frac{{\left( {X_{i,l} + X_{i,u} } \right)}}{{{\text{ivf}} \cdot V_{t} }} \cdot T} }}{{\frac{{2 \cdot \left( {X_{i,l} + X_{i,u} } \right)}}{{{\text{ivf}} \cdot V_{t} }}}}$$

The target concentration is given by:

5 $$\frac{dT}{{dt}} = k_{{{\text{syn}}}} - k_{\deg } \cdot T - \left( {k_{{\text{int}}} - k_{\deg } } \right) \cdot \frac{{\left( {1 - f_{u} } \right) \cdot X_{i,s} }}{{{\text{ivf}} \cdot V_{t} }}, \,{\text{initial}}\, {\text{condition}}\, T = T_{0}$$

The amount of residualized ^89^Zr after internalization of ^89^Zr-mAb is given by:

6 $$\frac{{{\text{d}}X_{r} }}{{{\text{dt}}}} = k_{{\text{int}}} \cdot \left( {1 - f_{u} } \right) \cdot X_{i,l} , \,{\text{initial}} \,{\text{condition}}\, X_{r} = 0$$

The total ^89^Zr concentration in the tumor is:

7 $$C_{t} = \frac{{X_{p,l} }}{{V_{p} }} \cdot {\text{pvf}} + \frac{{X_{i,l} + X_{r} }}{{V_{t} }}$$

The measured ^89^Zr uptake in SUV is:

8 $${\text{SUV}} = \frac{{C_{t} \cdot {\text{BW}}}}{{{\text{ID}}_{l} }}$$

Model simulations were performed using Berkely Madonna (version 8.3.18).

### Model performance

To evaluate the performance of the model we selected literature data on a ^89^-Zr labeled antibody against a well characterized target with measured plasma PK as well as tumor uptake as a function of time. Plasma PK as well as tumor uptake data for ^89^Zr-trastuzumab at an administered dose of 50 mg were obtained from literature [4]. Plasma PK data were digitized using Plot Digitizer (http://plotdigitizer.sourceforge.net/) and fitted to a bi-exponential curve using Matlab (version R2017b). The rate constants for exchange and clearance are calculated as outlined in [5] and given in Table 1. The sensitivity of model output to model parameters was assessed by evaluating the percentage change in model output with the change of model parameter by + 10% [2]. Parameters with a sensitivity smaller than 2% are not considered sensitive.

## Results

### Tumor uptake without HER2 expression as a function of time

**Fig. 3:**
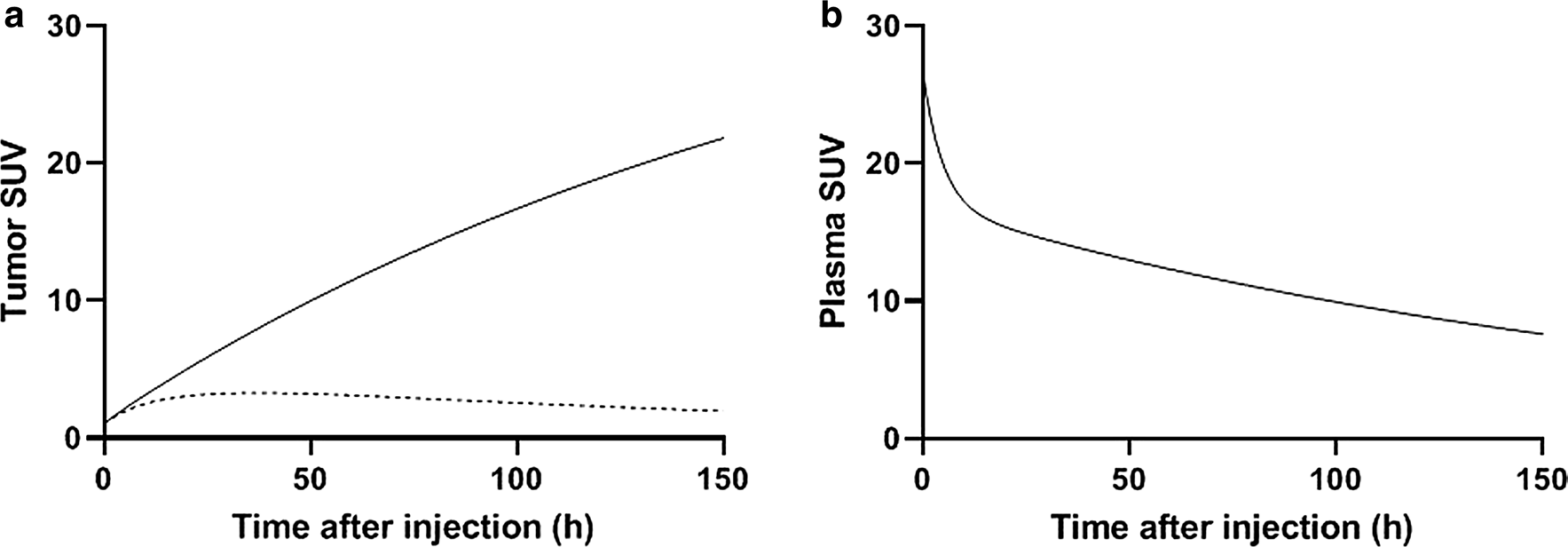
Tumor uptake (calculated) and plasma PK (from literature) of 50 mg ^89^Zr-trastuzumab as a function of time. Tumor uptake (**a**) for a tumor without target expression (dashed line) and a clinically HER2-positive tumor (IHC3) (solid line). Plasma PK (**b**)

Figure 3 shows the model derived tumor uptake of ^89^Zr-trastuzumab data for an administered dose of 50 mg (panel a; model ouput is based on the values in Table 1 with the intitial target concentration *T*_0_ = 0 nM, dashed line). For reference, also the plasma PK curve is given (panel b; not predicted in the model but used as an input function obtained from literature). Tumor uptake increases over time until plasma and interstitial concentrations are equal (Fig. 3b). From then on (typically after 24 h p.i.), tumor uptake decreases over time, proportional to the decrease in plasma concentration, since there is no driving force for extravasation (Fig. 1c). In case there is zero HER2 expression in the tumor, the model predicts a tumor SUV of ~ 2 at 120 h p.i., ~ 24% of the plasma SUV at this point in time (the sum of the vascular and mAb accessible interstitial volume fractions).

### Tumor uptake with HER2 expression as a function of time

For a clinically HER2-positive tumor (IHC3), tumor uptake increases over time, as no equilibrium is reached between the concentrations of unbound ^89^Zr-mAb in plasma and in interstitial space (Fig. 3a; model output is based on the values in Table 1, with *T*_0_ = 2700 nM, solid line). In this case, every mAb molecule entering the interstitial space will bind to a target (due to the abundance of free targets). Therefore, the interstitial concentration of unbound ^89^Zr-mAb is nearly zero and the driving force remains (Fig. 1d). The extravasation rate constant is the only parameter that impacts the predicted SUV at 120 h p.i., and a SUV of 19 is predicted at the value of 0.012 h^−1^ for the extravasation rate constant (see Table 1). The clinical study on ^89^Zr-trastuzumab from which the plasma PK data are taken reports a range in SUV from 2.5 to 20.2 [4] for HER2-positive tumors (all IHC3). This results in a range in extravasation rate constant from 0.0014 to 0.0128 h^−1^ (obtained by changing the value of this rate constant and keeping the other parameter values as given in Table 1).

### Tumor uptake as a function of HER2 concentration

**Fig. 4:**
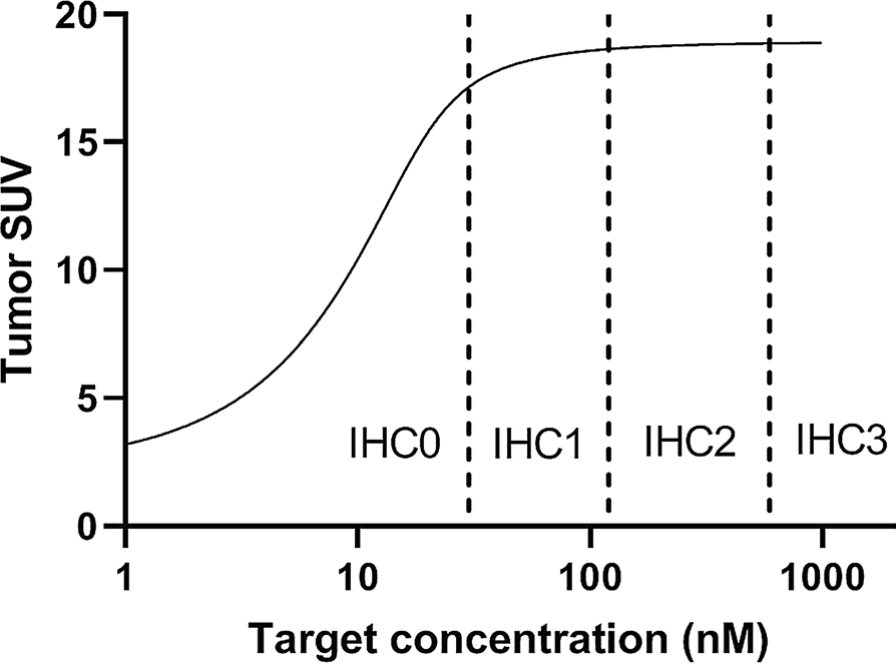
Tumor uptake of ^89^Zr-trastuzumab as a function of HER2 concentration. Total administered mass dose 50 mg. Vertical dashed lines indicate the boundaries between the IHC classes

At an administered dose of 50 mg, tumor uptake increases with increasing HER2 concentration from 1 to ~ 50 nM (Fig. 4; model ouput is based on the values in Table 1, with *T*_0_ ranging from 0 to 1000 nM). With increasing target concentration, the concentration of unbound antibody in the interstitial space decreases. Thus, the amount of mAb extravasating increases and results in a higher total uptake of mAb in the tumor. For target concentrations above 50 nM a plateau in SUV is reached, as there is an overload of targets for the amount of antibody administered (the concentration unbound mAb in the interstitial space is close to zero). The factor that determines the maximum tumor uptake is not the target concentration, but the extravasation rate constant.

## Discussion

^89^Zr-immuno-PET is a promising in-vivo tool to determine the presence of target expression and to quantify tumor uptake of ^89^Zr-labeled mAbs. Tumor uptake is determined by multiple factors, including mAb extravasation and target interaction.

Here we demonstrate the relation between tumor uptake and target concentration. Conceptually, a target-negative tumor can be distinguished from a target-positive tumor by ^89^Zr-immuno-PET, assuming other tumor characteristics are similar (Fig. 3). At a mass dose of 50 mg ^89^Zr-trastuzumab in a trastuzumab-naïve patient, we found that tumor SUV can distinguish between IHC0 and IHC1-2–3 (Fig. 4). This result may explain false-positive findings as described in literature, since tumor SUV cannot distinguish between IHC1-2–3.

So far, no clinical data is available for validation of tumor uptake for tumors that do not express the target. For HER2-negative tumors (for which limited HER2 expression cannot be excluded) a median SUV of 3.1 (range 2.0–5.7) was reported in literature [6]. Interestingly, HER2-negative tumors with hepatic localization showed a higher SUV than non-hepatic lesions (7.9 vs. 2.8). This may be due to a higher extravasation rate constant for tumors located in the liver.

When considering the use of a SUV threshold to assess target status it is important to realize that the rate of extravasation is the key factor that determines the maximum tumor uptake. This tumor characteristic is expected to vary between and within patients (e.g., for tumor type and localization). In addition, as plasma clearance increases, e.g., due to the presence of an antigen sink [7], uptake will be lower due to the lower concentration of mAb in plasma. Published plasma PK curves at 1 mg/kg and 8 mg/kg can be used to explore the effect of an increase in total mass dose on tumor uptake [8]. At 120 h p.i., SUV is predicted to increase from 21 to 26. Although the absolute amount of mAb in the tumor is much higher in the latter case, this is not reflected in the SUV as this uptake value relates to labeled mAb only.

Further experimental validation of the mathematical model will be possible with increasing availability of total-body PET scanners, mainly through their ability to better define tumor uptake as a function of time.

The general framework designed in this study can be applied to improve future clinical ^89^Zr-immuno-PET studies. For a novel antibody with different target kinetics, the relation between SUV and target concentration can be predicted by inserting the corresponding target parameters in the model.

## Conclusion

We demonstrated how tumor uptake as assessed by ^89^Zr-immuno-PET is related to the target concentration, using mathematical modeling of ^89^Zr-labeled antibody disposition in the tumor.

The example for ^89^Zr-trastuzumab illustrates the potential to assess target status. The pitfall of false-positive findings depends on the target concentration used to clinically define target positivity and on the administered mass dose.

## Data Availability

All data generated and analysed in this study are included in this published article.
